# EU-funded research to advance agroecological weed management in Europe, Part I: vision

**DOI:** 10.12688/openreseurope.20296.1

**Published:** 2025-08-01

**Authors:** Alexandros Tataridas, Maria Anastasi, Yedra Vieites-Álvarez, David López-González, Claudia Campillo-Cora, Alessandro Giusti, Adela María Sánchez-Moreiras, Helena Freitas

**Affiliations:** 1 Department of Life Sciences, Centre for Functional Ecology: Science for People and the Planet (CFE), Associate Laboratory TERRA, Department of Life Sciences, University of Coimbra, Coimbra, Coimbra District, 3000-456, Portugal; 2 Cyprus Research and Innovation Center (CYRIC), Nicosia, 2643, Cyprus; 3 Universidade de Vigo. Departamento de Bioloxía Vexetal e Ciencia do Solo, Facultade de Bioloxía, Vigo, Vigo, 36310, Spain; 4 Instituto de Agroecoloxía e Alimentación (IAA), Universidade de Vigo, Campus Auga, Ourense, Ourense, 32004, Spain; 5 Departamento de Bioloxía Vexetal e Ciencias do Solo, Área de Edafoloxía e Química Agrícola. Facultade de Ciencias de Ourense, Universidade de Vigo, As Lagoas s/n, Ourense, Ourense, 32004, Spain

**Keywords:** agroecology, agroecological transitions, herbicides, crop diversification, weed management

## Abstract

Weeds are a major challenge in agriculture, significantly reducing crop yields, threatening food security, and causing adverse effects on ecosystems, human health, and biodiversity. Although synthetic herbicides have been the primary method of weed control and remain widely used due to their effectiveness, growing concerns about their environmental and health impacts—combined with regulatory pressures in the European Union to reduce chemical inputs—have increased the demand for alternative strategies. **Agroecological Weed Management** (AWM) has emerged as a sustainable strategy, integrating ecological principles and combinations of diverse practices (such as *crop rotation, cover cropping, digital tools and mechanical methods*) to balance productivity with environmental conservation. AWM emphasizes the co-design of solutions with stakeholders across the agri-food value chain, prioritizing non-chemical approaches that enhance biodiversity, improve soil health, and leverage the ecosystem services provided by plants. This paradigm shift aligns with European Union targets to drastically reduce pesticide use in the coming years and promote sustainable farming practices, while keeping EU agriculture competitive. However, **transitioning to AWM requires addressing challenges** such as economic pressures on farmers, overreliance on herbicides, and the need for education-training, extension services and innovative low-cost technologies. Through research on AWM approaches, **three European-funded projects aim to present their collective vision for sustainable weed management in Europe**, serving as the foundation for a series of articles that will document their methodologies, findings, and progress throughout their implementation.

## Plain language summary

The article “EU-funded research to advance agroecological weed management in Europe, Part I: vision” is the first part of a series of planned articles that will be published by the European-funded projects GOOD, CONSERWA and AGROSUS. This initial piece outlines the shared vision of the coordinating teams and presents the methodologies adopted across the three projects to develop, test, assess, demonstrate, and promote agroecological weed management with the overarching goal of reducing reliance on herbicides in Europe.

## Disclaimer

*The views expressed in this article are those of the author(s). Publication in Open Research Europe does not imply endorsement of the European Commission*.

## Introduction

Weeds are plants classified as crop pests that directly affect crop productivity leading to significant crop yield reductions, threatening food security, and adversely affecting the animals, humans, and environment due to ecosystem disservices. This characterization has been the dominant theory of the last decades in the rural sector in Europe. However, in recent years, current challenges on the sustainability of agricultural systems and holdings and the need to shield them against climate change have triggered a different concept, that of ecological management of weeds ( Gazoulis *et al.*, 2024). Weed management stands as one of the foremost challenges in agriculture nowadays, impacting both conventional and organic, as well as regenerative, farming systems. For decades, synthetic herbicides have been invaluable tools for farmers in controlling weeds. However, the short and long-term impacts of herbicide use on animals, soil health, pollinators, and human health necessitated actions towards reducing or eliminating their use, underscoring the urgent need for alternative solutions that are affordable, easy-to-adopt and effective at the same time. Below is an overview of the vision of the coordinating teams of three EU-funded projects focused on ecological and sustainable weed management, along with the approach and methodology for mainstreaming research and the adoption of Agroecological Weed Management (AWM).

## Synthetic pesticides: a topic of polarization in the European Union

Certain active substances of pesticides have been found to contaminate groundwater, affect soil quality and health, and leave residues in food products, raising significant concerns about food safety and their environmental impact. The repeated application of these chemicals increases the risk of toxicity and negatively impacts biodiversity, adversely affecting various natural weed enemies. Nevertheless, synthetic herbicides such as glyphosate remain a significant tool for EU farmers ( Neve *et al.*, 2024), while alternatives are being tested and assessed on the ground for their efficacy, applicability and benefits. Economically, the burden of weed management and overall crop protection poses a significant challenge for farmers, particularly in regions with biological invasions ( Tataridas *et al.*, 2022a), herbicide-resistant and noxious weeds or where there is an effort to reduce fuel consumption by minimizing mechanical interventions. The costs associated with weed management have surged in recent years, rendering herbicide use an expensive process that does not always guarantee satisfactory outcomes or profitability. Additionally, the growing resistance of weeds to certain herbicides is an escalating problem, diminishing the effectiveness of treatments and necessitating higher doses or the development of new products if farmers continue to rely solely on spraying as their weed control strategy. Compliance with various European regulations and targets, such as (1) the 50% reduction of pesticides by 2030, (2) the increase in land under organic farming, and (3) the reduction of pollutants and greenhouse gas emissions, further pressures farmers to seek more sustainable solutions. International regulations advocate for decreased chemical usage while promoting the adoption of environmentally friendly alternative weed management methods. This shift towards more ecological weed management necessitates new strategies, education, information dissemination, and innovation. However, this ecological transition is not always and everywhere acceptable in the continent, while several events and decisions have formed a controversial and polarized scenery. In particular, such polarizing issues (which will not be commented on in this article) include the widespread protests by EU farmers in 2024, the renewal of the registration of glyphosate use for another ten years in 2023, various reactions to the implementation of the EU Green Deal, etc. In conclusion, the paradigm of weed management is shifting from reliance on chemical herbicides to the adoption of more sustainable, ecological practices. The challenge lies in balancing effective weed management with environmental conservation and “one health” considerations. This transition calls for a concerted effort in research, policy-making, and practical application to ensure that future agricultural systems are both productive and sustainable.

## Transitional research for weed science: from conventional to agroecological

**Figure 1.**
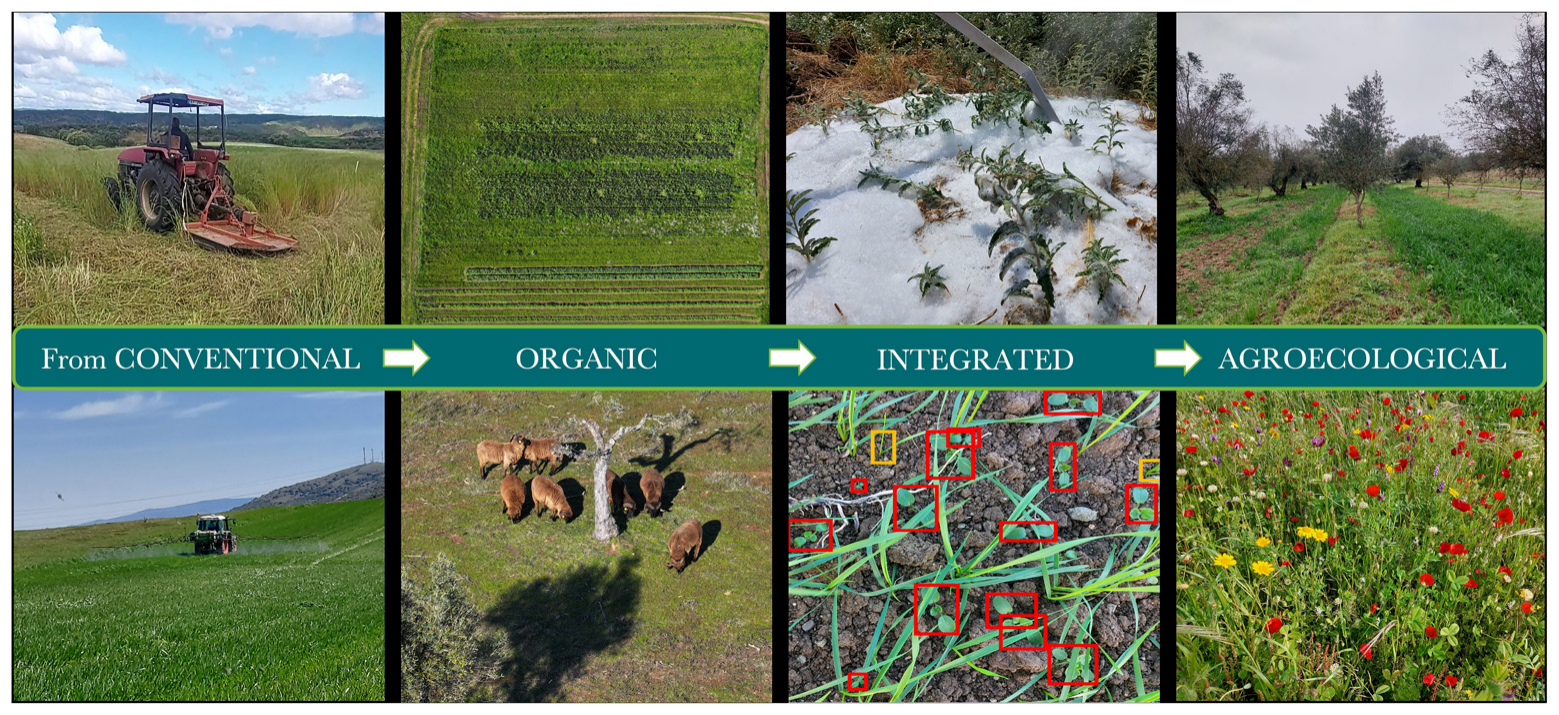
Weed management practices from conventional to agroecological. Copyrights: Alexandros Tataridas, University of Coimbra.

Acknowledging that the era of weed control has transitioned into the era of weed management, it is clear that many farmers have adopted new perspectives and agricultural practices, especially in the areas of pest management and crop protection. Currently: (1) **crop rotation, cover cropping** and **intercropping** have become desirable practices, offering economic diversification and being implemented in an increasing number of regions and agricultural systems; (2) year-round **soil coverage** with plants significantly meets nitrogen needs, maintains and improves soil fertility, aids in water retention—crucial under drought conditions—and suppresses weed growth if residues are left on the soil after cover crop termination; (3) systematic tillage is now viewed as a practice that contributes to soil erosion, prompting the search for technological and mechanical solutions that **minimize soil disturbance**, particularly in the upper soil layers; (4) **reducing fuel consumption**, which entails reducing the use of agricultural tractors, is a pressing necessity; (5) more farmers and cooperatives seek to be **educated and trained in new, cost-effective practices, technologies and services** that are equally effective; (6) chemical inputs (primarily referring to pest management) are no longer seen as a panacea, as the **direct and indirect impacts on operators, soil, water, and biodiversity**—particularly the effects on non-target organisms and pollinators, and their residue in some cases—are evident; (7) **economic uncertainty and the rising prices** of products, resulting from market instability, conflicts, and the effects of climate change, reduce farmers' dependence on the chemical market and drive developments towards more **independent, cooperative, and sustainable agricultural schemes** that primarily utilize non-chemical methods and regenerative practices to remain competitive; (8) the idea that some **minor yield losses due to weed competition are acceptable** is gaining traction, as it reduces the costs of weed management, prioritizing actions when and only if needed; (9) **consumer markets are increasingly prioritizing quality over quantity**, favoring food products from organic, regenerative, and agroecological farms, recognizing the importance of inspections, certifications, and labeling; and (10) the perception that **not all weeds are harmful to crops** is spreading, and in some cases, even their presence in the agroecosystem should be encouraged and utilized ( Figure 1).

The modern approach to weed management has evolved to embrace a more comprehensive and sustainable outlook. Farmers are progressively implementing practices that incorporate ecological principles, striving to achieve a balance between agricultural productivity and environmental conservation ( HLPE, 2019; Tataridas *et al.*, 2022b). This shift is evident in the growing adoption of diverse cropping systems and the conscious reduction of chemical inputs, reflecting a broader dedication to preserving long-term soil health and biodiversity. Concurrently, the agricultural economic landscape is experiencing a significant transformation. This change is propelled by increasing market demand for sustainably produced food and the necessity for resilient farming systems capable of withstanding certain challenges. The ongoing transition highlights the critical need for continuous farmer education, advancements in agricultural technology, and supportive policy frameworks ( Tataridas & Freitas, 2024). These elements are essential in promoting sustainable weed management practices rooted in agroecological principles that effectively address current environmental concerns and economic realities in agriculture.

## The concept of agroecological weed management

Although there is no clear and universally agreed definition, agroecological weed management encompasses a range of practices based on ecological principles that prioritize environmental sustainability and consider the well-being and health of both humans, ecosystems, plants and animals in the decision-making processes on weed management ( Tataridas *et al.*, 2023). These practices encompass a combination of deterrent, preventive, and interventive measures and methods (including natural, cultural, traditional, biological, mechanical, technological, and precision approaches) aimed at reducing the impact of weeds on agroecosystems. They rely on creating complex yet functional agroecosystems with increased diversity and complexity, akin to natural ecosystems undisturbed by human interference. The goal is to enhance the resilience of agricultural, agroforestry, and agro-pastoral systems against weeds by leveraging crop competitiveness, disrupting the biological cycle of weeds, and implementing alternative, primarily non-chemical, weed management methods where and when necessary ( Deguine *et al.*, 2023).

The joint definition of the three coordination teams about agroecological weed management is the following. This definition is not necessarily adopted by all partners within each consortium, as they may work with or present different interpretations of AWM:

*Agroecological Weed Management (AWM) is a collaborative approach that involves all actors across agri-food value chains (from farmers to consumers) in co-designing ecological, cultural, and technological solutions to manage weeds by diversifying landscapes and agricultural systems, supported by sustainable and transformative policies that aim to prevent weed infestation, reduce weed pressure, limit dependence on herbicides and potentially utilize the ecosystem services of weed species, to reverse biodiversity declines and safeguard food security.*

**Figure 2.**
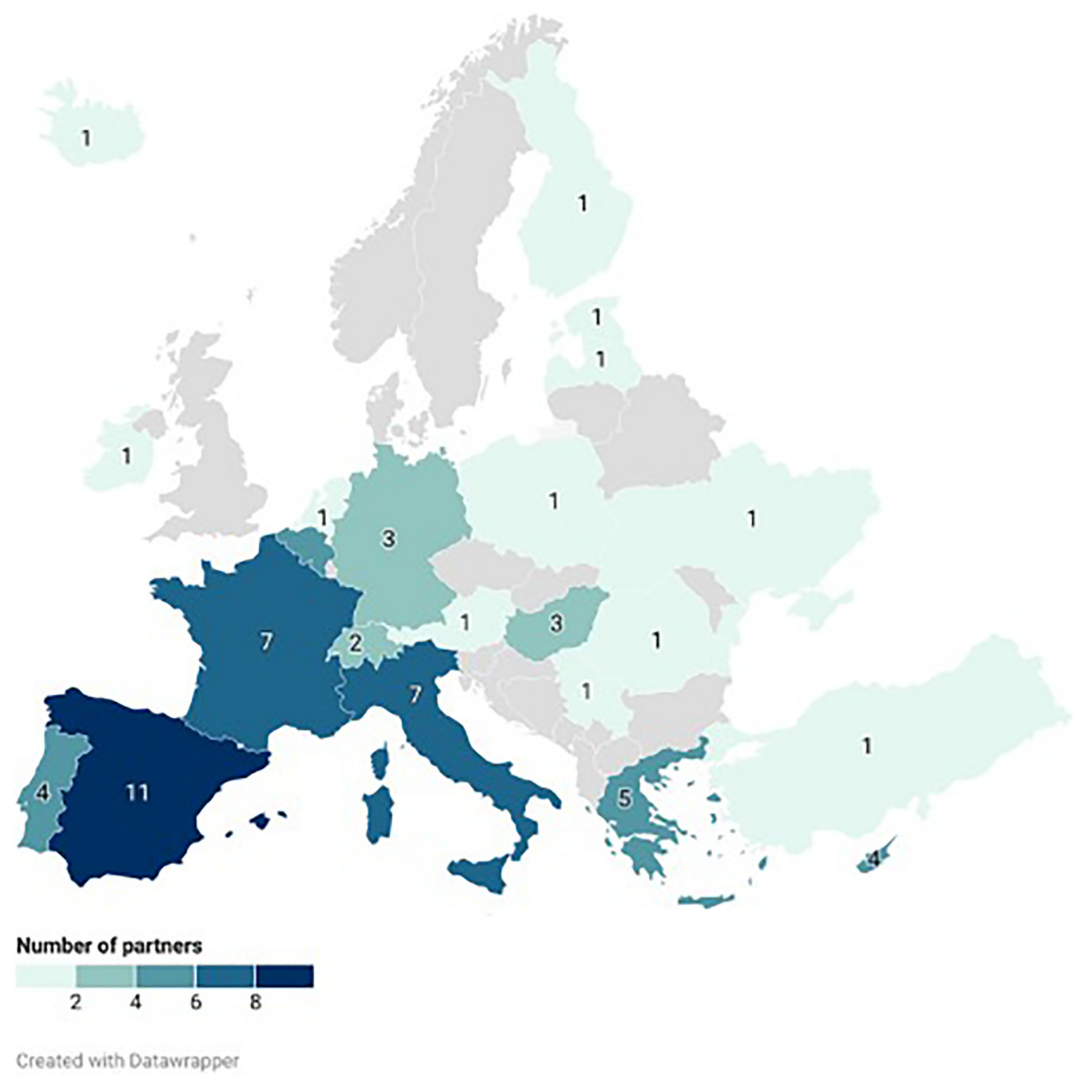
Geographical distribution of partners in GOOD, AGROSUS, CONSERWA. The map was created with Datawrapper. In accordance with the European Union’s designation which is without prejudice to positions on status, and is in line with UNSCR 1244/1999 and the ICJ opinion on the Kosovo declaration of independence.

The three sister projects test AWM practices in various farming systems across the EU and Associated Countries ( Figure 2).

### The GOOD project

**Figure 3.**
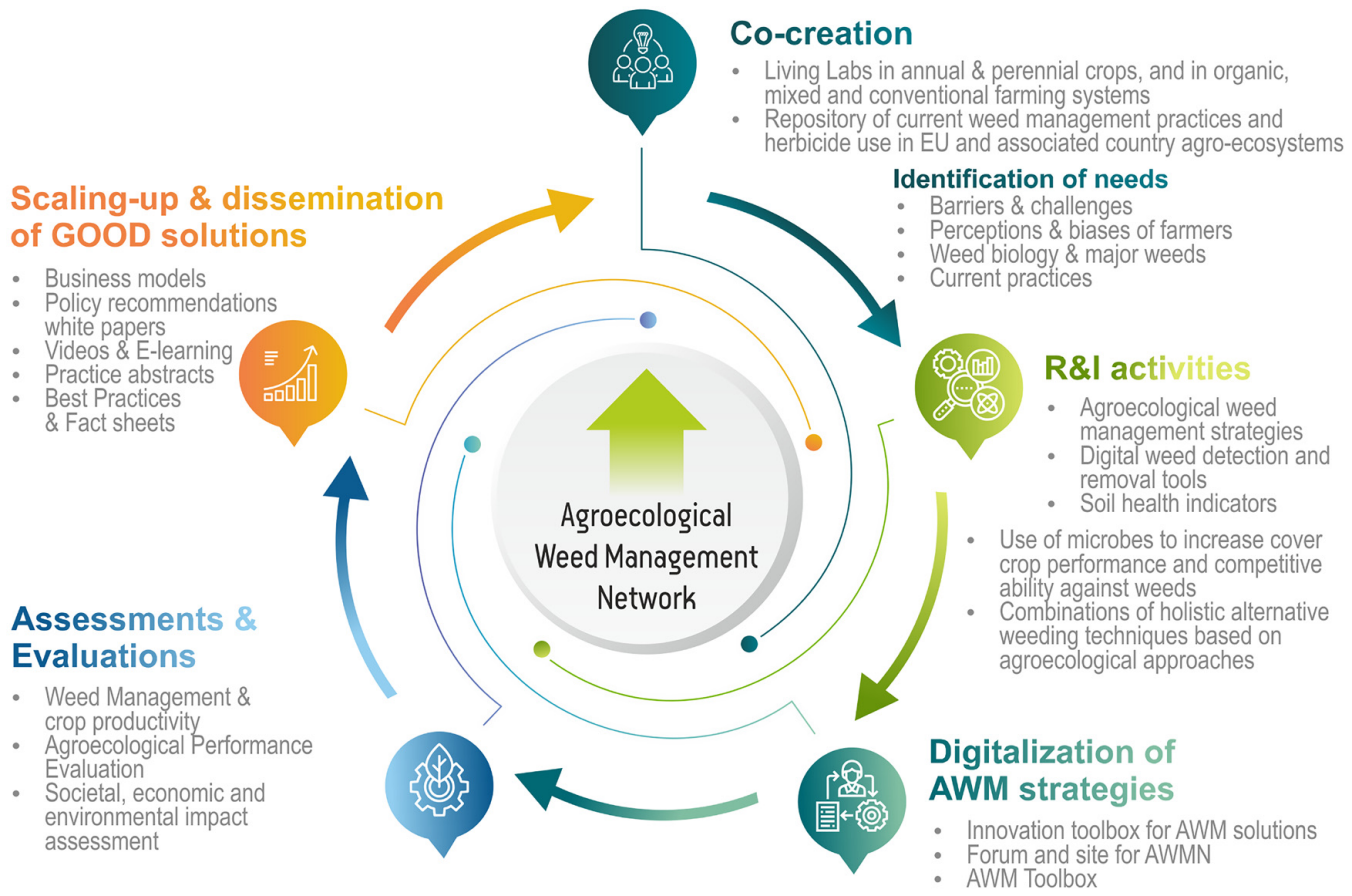
Methodology of GOOD. This figure 3 has been reproduced with permission from the coordinator of GOOD.

The GOOD project ( https://www.goodhorizon.eu/) "Agroecology for Weeds," is a European project addressing the challenges of weed management in diverse agricultural systems. In response to the demand for reducing chemical inputs (herbicides) in agriculture, the project operates within a multi-disciplinary, stakeholder-engaged framework. It integrates knowledge from social sciences, digital technologies, and natural sciences to co-create, test, validate, and demonstrate sustainable weed management practices based on agroecological strategies tailored for conventional, organic, and mixed farming systems across Europe. A central element of the GOOD project is the establishment of 16 Living Labs (LLs) in nine countries and the creation of an Agroecological Weed Management Network (AWMN) ( Figure 3). These platforms serve as collaborative spaces for various stakeholders, including farmers, advisors, consumers, researchers, policymakers, and industry actors. The LLs test and evaluate combinations of AWM strategies through real-world experimentation and co-creation processes. Key strategies include the use of cover crops, innovative solutions such as drones for weed mapping, and native arbuscular mycorrhizal fungi (AMF) inoculants. These strategies aim to improve the competitiveness of crops and cover crops against weeds while promoting soil health and ecological balance. GOOD also explores the socio-economic and policy dimensions of weed management. By examining behavioral drivers, market acceptance, and the environmental impact of AWM practices, the project aims to build a robust sustainability assessment framework. This includes developing business models and policy recommendations to support the widespread adoption of agroecological practices. Through dissemination, training programs, and stakeholder engagement, GOOD seeks to inspire a shift in perceptions and practices, facilitating an agroecological transition that benefits both people and nature. Its outcomes are scalable and transferable, ensuring long-term impact across European agri-food systems. The project also aims to enhance trust in AWM strategies by demonstrating tangible benefits and releasing a digital Decision Support System, the AWM Toolbox, to aid in weed management decisions.

***Results beyond the state-of-the-art***

GOOD has advanced AWM, offering sustainable solutions to reduce herbicide dependence and build resilient agricultural systems. A major achievement is the practical use of native arbuscular mycorrhizal fungi inoculants, adapted for seed coating to enhance weed suppression and soil health. This innovation will reduce herbicide reliance while improving resilience across farming systems. The project also leverages digital technologies such as drone-based weed mapping. A Decision Support System will offer tailored recommendations for cover crops and combinations of AWM strategies. Central to GOOD’s success is the establishment of Living Labs, which foster collaboration among farmers, researchers, and other stakeholders to co-create and validate AWM solutions. This participatory framework ensures practical, widely accepted strategies. More than 900 stakeholders participated in questionnaires, and over 480 were interviewed to gather insights into their perceptions and needs regarding AWM. A digital AWM repository on current and agroecological weed management practices is already available ( https://www.goodhorizon.eu/platform/description/). Baseline assessments of soil health and biodiversity, paired with Life Cycle Assessments, provide a foundation for evaluating the environmental, economic, and social impacts of AWM. These metrics will highlight the benefits of transitioning to sustainable practices. GOOD’s outcomes promise far-reaching impacts: reducing herbicide use and promoting biodiversity to improve ecosystems, offering cost-effective farming solutions, and empowering stakeholders through inclusive approaches. To accelerate adoption, continued research, financial incentives, affordable tools, and supportive policies are crucial. Expanding international collaborations will further drive the adoption of AWM innovations.

### The AGROSUS project

**Figure 4.**
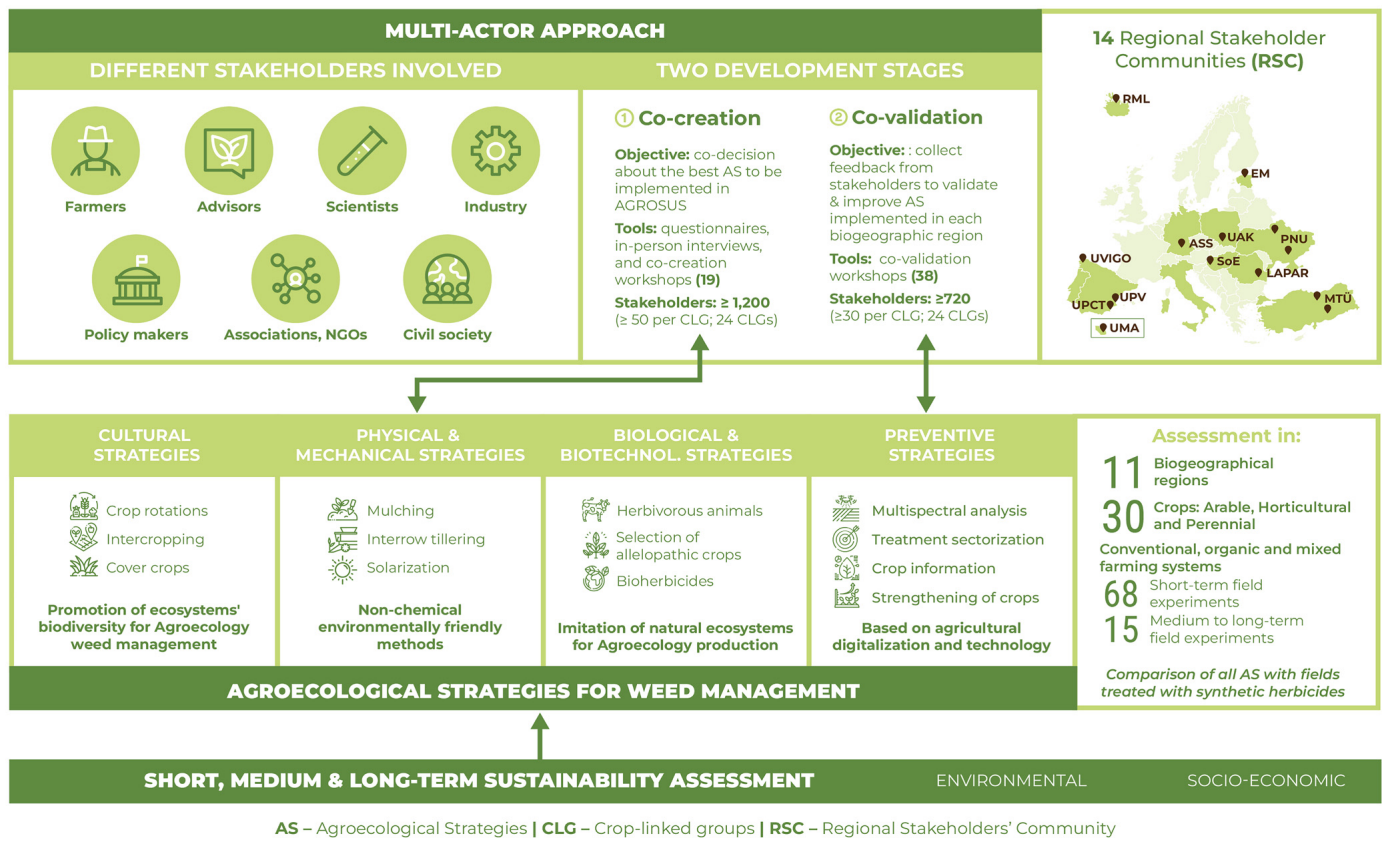
Methodology of AGROSUS. This figure 4 has been reproduced with permission from the coordinator of AGROSUS.

The AGROSUS project (AGROecological strategies for SUStainable weed management, www.agrosus.eu) is a European initiative that aims to promote AWM methods through agroecological innovations. In response to growing environmental and societal concerns over herbicide dependence, AGROSUS integrates traditional knowledge, scientific research, and technological development in a participatory, multi-actor framework. The project focuses on the development, implementation, and assessment of locally adapted AWM practices across diverse agro-climatic zones in Europe. AGROSUS aims to reduce herbicide inputs, enhance biodiversity, and improve soil and crop health by promoting sustainable, farmer-driven solutions. A central pillar of AGROSUS is its use of on-farm experimentation, including the co-design and testing of alternative AWM strategies in collaboration with farmers, advisors, scientists, and policy stakeholders. These activities are conducted across 24 living labs in 11 countries, providing a wide range of agronomic, ecological, and socio-economic contexts ( Figure 4). AGROSUS fosters strong stakeholder involvement through workshops, field visits, and innovation platforms, allowing the exchange of ideas, experiences, and best practices. Strategies explored include mechanical weeding, mulching, competitive crop varieties, crop rotations, and intercropping systems, all grounded in agroecological principles. In addition to traditional techniques, AGROSUS is also developing and testing new agricultural technologies such as robots for precision and non-chemical weed control—bringing cutting-edge solutions into agroecological practice. These practices are selected and adapted in a bottom-up process, ensuring they are regionally relevant, economically viable, and socially acceptable. The project also emphasizes the development of sustainability indicators and decision support tools to evaluate and guide the adoption of these practices. By examining both the environmental impact and socio-economic implications, AGROSUS provides a robust framework for transitioning to low-input systems. Its goal is not only to offer alternatives to herbicides, but to embed these practices into broader farm management and policy frameworks. Communication, education, and training play a key role, with dedicated efforts to disseminate outcomes through open-access platforms, policy briefs, farmer guides, and stakeholder events. The project aims to contribute to long-term resilience, knowledge exchange, and the mainstreaming of AWM across Europe and beyond.

***Results beyond the state-of-the-art***

AGROSUS advances the field of AWM by delivering innovative, science-based, and farmer-led solutions designed to meet the pressing need for sustainable alternatives to herbicide use. Through a participatory, multi-actor approach, the project has co-developed and validated AWM strategies across more than 70 experimental units in 11 countries, ensuring each solution is finely tuned to local agronomic, climatic, and socio-economic conditions. This approach increases both the practical relevance and the likelihood of adoption by farmers on the ground. Among the most notable achievements of the project is the development of a database ( https://agrosus.eu/weeds-database/) with valuable information (name, cycle, type, control method, languages...) on more than 100 weeds for their identification and effective agro-ecological treatment and that is linked to GOOD and CONSERWA projects. Alongside this, AGROSUS has advanced the use of mechanical weeding tools, fine-tuned for a variety of farming systems, showing strong results in reducing weed pressure and input costs—all without compromising yields. A key technological breakthrough is the design and testing of advanced agricultural robots, capable of precision weed management without relying on chemical inputs. These autonomous tools are a leap forward for sustainable agriculture, offering labor-saving, environmentally friendly alternatives that can adapt to different field contexts. Complementing these mechanical innovations, AGROSUS is also developing a new botanical-based bioherbicide, providing a natural solution to weed control with the potential to further minimize environmental impacts. To support evidence-based adoption, AGROSUS pioneers the integration of qualitative and quantitative sustainability indicators, encompassing ecological, economic, and social dimensions. These indicators feed into a robust evaluation framework that helps farmers, advisors, and policymakers assess the performance, feasibility, and impacts of different AWM strategies. AGROSUS’s participatory research model is reinforced by its deep stakeholder engagement: over 900 farmers and more than 400 stakeholders—including advisors, researchers, policymakers, industry actors, and NGOs—have contributed to shaping, testing, and assessing project solutions. This inclusive process builds trust and ownership, encourages uptake, and helps cultivate a Europe-wide innovation ecosystem committed to sustainable weed management. The long-term ambition of AGROSUS is to spark a paradigm shift in weed management—moving away from chemical dependence and toward diversified, resilient, and ecologically sound practices. Its outcomes promise wide-reaching benefits: enhancing biodiversity and soil health, ensuring cost-effective farming, and supporting public health and environmental sustainability. Through its integrative, scalable approach, AGROSUS lays the groundwork for a future of agriculture that is more robust, more regenerative, and more in harmony with both nature and society.

### The CONSERWA project

**Figure 5.**
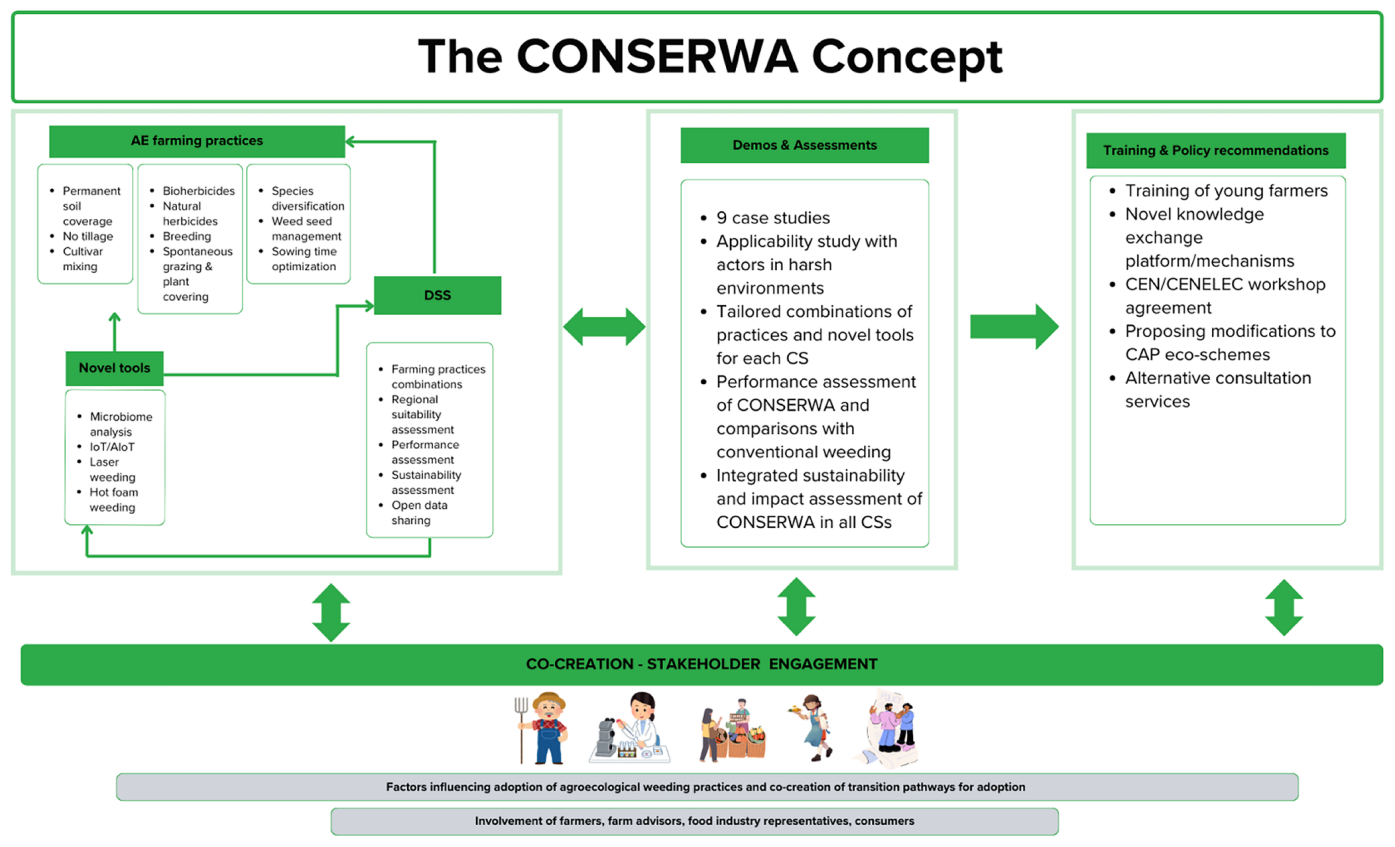
Methodology of CONSERWA. This figure 5 has been reproduced with permission from the coordinator of CONSERWA.

**CONSERWA** (Evidence-based support for transition to agroecological weed management in diverse farming systems and European regions, https://conserwa.eu/) is a European funded project designed to addresses sustainable weed management challenges through a multi-actor, evidence-based approach. The project leverages on the principles of agroecology in combination with technological tools and methods applied in nine case studies across Europe. Sustainable weed management is an urgent priority in the transition to resilient and environmentally sound agricultural systems. While agroecology offers a transformative framework for achieving this, its adoption -particularly for weed control- still remains limited despite well-established benefits as several key challenges hinder progress. First, while agroecological practices are applied to varying degrees, their optimal combinations and context-specific implementation are not well understood. Second, adoption is constrained by weak policy support, limited market incentives, and insufficient social infrastructure to guide farmer decision-making. Third, climate change is expected to reduce the effectiveness of chemical herbicides, yet its implications for non-chemical, agroecological weed management remain underexplored. CONSERWA assesses and optimizes combinations of agroecological practices across diverse European agro-climatic regions and evaluates their performance under current and future climate scenarios using advanced tools ( Figure 5). Promotes stakeholder learning and decision-making via an open-access Decision Support System and engages with value chain actors to understand and influence farmer adoption dynamics. Through extensive case studies, policy engagement, and farmer training, CONSERWA aims to unlock the full potential of agroecology for sustainable weed management in Europe.

***Results beyond the state-of-the-art***

CONSERWA advances the state-of-the-art (SotA) on agroecology by strategically integrating various methods and tools tailored to specific scenarios. Notably, it pioneers incorporation of climate change considerations, aiming to predict the efficacy of agroecological practices and novel tools under climate-impacted conditions, and to develop adaptive strategies accordingly. To ease the adoption of agroecological weed management (AWM) among farmers and increase acceptance across the value chain, CONSERWA introduces an advanced, open-access Decision Support System (DSS). It will guide users in selecting optimal combinations of agroecological practices based on contextual factors addressing known adoption challenges, such as the complexity of ecological weed management design. It also aims at enabling performance and sustainability assessments of ongoing practices through data-driven metrics, aiding policymakers in evaluating current strategies and designing effective Common Agricultural Policy (CAP) eco-schemes, and promote stakeholder collaboration by facilitating knowledge exchange and providing open access to valuable public datasets. The project also introduces new methods and indicators which will be embedded within the DSS to evaluate intervention outcomes in the CS. Furthermore, by conducting field trials and case studies in diverse agro-climatic zones and crop systems, CONSERWA provides robust, real-world evidence on the effectiveness and scalability of agroecological interventions. This multi-site demonstration approach ensures that findings are not only scientifically validated but also relevant to a broad spectrum of farming conditions within Europe, highlighting the flexibility and adaptability of agroecological models across different geographies and farming systems. CONSERWA innovatively combines modern technologies—such as remote sensing, data analytics, machine learning, and automated monitoring tools—with traditional agroecological knowledge. This synergy enhances precision, efficiency, and impact of interventions, supporting sustainable pest management, soil health improvement, and biodiversity enhancement. The result is a set of practical, scalable solutions that bridge the gap between traditional ecological farming and high-performance modern agriculture. A key strength of the project lies in its multi-actor approach, fostering strong partnerships across the agricultural value chain. By engaging with farmers, cooperatives, agribusinesses, policymakers, certification bodies, and standardization organizations, CONSERWA ensures that agroecological solutions are not only scientifically robust but also economically viable, socially accepted, and policy relevant. This collaboration is essential to co-create enabling environments that facilitate agroecological innovation and adoption at scale. Recognizing the critical role of education and capacity building, CONSERWA places significant emphasis on training programs tailored to young farmers, agricultural advisors, and students. These initiatives include hands-on field demonstrations, digital learning resources, and participatory workshops designed to build the competencies needed for agroecological transition. By investing in future practitioners, the project ensures the continuity and long-term sustainability of its outcomes. Effective agroecological transformation requires transparent, timely, and interoperable data flows. CONSERWA facilitates this through the creation of open-access data platforms and collaborative knowledge-sharing mechanisms. These tools allow stakeholders to access, reuse, and contribute to high-value public datasets, enabling evidence-based decision-making and fostering innovation. Enhanced data exchange also supports monitoring and evaluation processes, ensuring that progress is tracked and communicated effectively across the value chain.

## Conclusions

Agroecological weed management is an increasingly prominent field of both theoretical and applied research in Europe and beyond. It is rooted in the principle of reciprocity among farmers, researchers, and other stakeholders across agri-food value chains, creating a ripple effect that supports the agroecological and sustainable transformation of food systems. The serendipity of receiving funds for transnational research reflects the EU’s priorities in environmental protection, and biodiversity conservation and restoration. It also creates a stratum for implementing EU policies and laws, such as the new Nature Restoration Regulation. The three sister projects (GOOD-AGROSUS-CONSERWA) are working collaboratively to propel the transition toward sustainable weed management based on agroecological principles bringing a multitude of benefits to farming systems, agroecosystems, local communities and policy making. This first joint publication is the contour of actions that will be deployed in the next few years. These actions will generate coherent practical recommendations to farmers, spearhead debates on pesticides use reduction and accentuate risk-tolerant policy recommendations aligned with the evolving priorities of the upcoming Common Agricultural Policy (CAP) post 2027. At least two more articles are planned, one aggregating and presenting research findings (Part II: development) and one outlining future directions (Part III: legacy).

## Ethics and consent statement

Ethical approval and consent were not required

## Data Availability

No data or software are available.
