# Supplementary material for: EU-funded research to advance agroecological weed management in Europe, Part I: vision
Source: Open Res Eur. 2025 Aug 1;5:211. [Version 1] doi: 10.12688/openreseurope.20296.1 (PMC12780524; doi:10.12688/openreseurope.20296.1)
Supplement: Peer review history

# Reviewer report: Reviewer response for version 1

**DOI:** 10.21956/openreseurope.21960.r65552

**Published:** 2026-01-07

**Authors:** EMANUELE RADICETTI

**Affiliations:** 1 University of Ferrara, Ferrara, Italy

Dear Editor and dear authors,

 I carefully reviewed the manuscript entitled: EU-funded research to advance agroecological weed management in Europe, Part I: vision". 

 The article is submitted as Open Letter to be used as introductory section showing the progress of the following Horizon Europe projects: **GOOD, AGROSUS, and CONSERWA**.

 The manuscript addresses the critical challenge of moder agroecosystems in a context of a transition of European agriculture. The concern is related to the heavy reliance on synthetic herbicides and the prespective is driven by the European Green Deal’s target of a 50% reduction of herbicides by 2030.

 The authors analyze the vision for **Agroecological Weed Management (AWM)** based on the methodology of co-creation and "Living Labs". It is reported how the above-mentioned projects collaborate to develop, test, and deploy AWM solutions throughout the adoption of a range of solution such as cover cropping, mechanical weeding and digital decision support systems. The involvment of farmers, policymakers, and industry stakeholders should overcome economic and social barriers to adoption.

 The article is scientifically sound and meets the criteria for an Open Letter. It accurately describes the intent and methodology of the research projects without making unsupported claims about their results.

 In my opinion, the manuscript provides a clear, well-referenced, and balanced vision for the future of agroecological weed management research in Europe.

Where applicable, are recommendations and next steps explained clearly for others to follow? (Please consider whether others in the research community would be able to implement guidelines or recommendations and/or constructively engage in the debate)

Partly

Does the article adequately reference differing views and opinions?

Yes

Are all factual statements correct, and are statements and arguments made adequately supported by citations?

Yes

Is the rationale for the Open Letter provided in sufficient detail? (Please consider whether existing challenges in the field are outlined clearly and whether the purpose of the letter is explained)

Yes

Is the Open Letter written in accessible language? (Please consider whether all subject-specific terms, concepts and abbreviations are explained)

Yes

Reviewer Expertise:

Agronomy

I confirm that I have read this submission and believe that I have an appropriate level of expertise to confirm that it is of an acceptable scientific standard.

# Reviewer report: Reviewer response for version 1

**DOI:** 10.21956/openreseurope.21960.r58262

**Published:** 2025-09-04

**Authors:** Vesna Dragičević

**Affiliations:** 1 Maize Research Institute “Zemun Polje”, Belgrade, Serbia

Open letter provides information about agroecology position nowadays, why the shift from the commonly used herbicide strategy to eco-friendly weed management is required, including the possible models of agroecology integration into agricultural production and society, in general.

 Three EU funded projects offer reliable solutions for the agroecology implementation. Irrespective of the same goals, each of them covers some different viewpoints and aspects, including various practices and tools for eco-friendly weed management. While GOOD and AGROSUS are focused on concrete solutions in real-time, including their implementation on farmer’s fields, as a part of transition, supported by policy transformation, CONSERWA takes into account climate change, comparing various scenarios. All three Projects work together to provide reliable solutions, which could be rapidly used and implemented through platforms for agroecological weed management.

 Suggested modification: **Chapter “Synthetic pesticides: a topic of polarization in the European Union”** – the last 16 lines are more focused on paradigm of alternative solutions, not on herbicide problem, by itself, thus it is recommended to relocate and adapt some parts of the text as introductory of the next chapter: **Transitional research for weed science: from conventional to agroecological.**

Where applicable, are recommendations and next steps explained clearly for others to follow? (Please consider whether others in the research community would be able to implement guidelines or recommendations and/or constructively engage in the debate)

Yes

Does the article adequately reference differing views and opinions?

Yes

Are all factual statements correct, and are statements and arguments made adequately supported by citations?

Yes

Is the rationale for the Open Letter provided in sufficient detail? (Please consider whether existing challenges in the field are outlined clearly and whether the purpose of the letter is explained)

Yes

Is the Open Letter written in accessible language? (Please consider whether all subject-specific terms, concepts and abbreviations are explained)

Yes

Reviewer Expertise:

Cropping technology, soil science, agroecology, crop quality, crop physiology.

I confirm that I have read this submission and believe that I have an appropriate level of expertise to confirm that it is of an acceptable scientific standard.

## References

[1] : EU-funded research to advance agroecological weed management in Europe, Part I: vision. *Open Research Europe* .2025;5: 10.12688/openreseurope.20296.1 10.12688/openreseurope.20296.1 40386579
